# Automated seizure detection using wearable devices: updated systematic review and meta-analysis of tonic-clonic and focal seizure detection

**DOI:** 10.3389/fbioe.2026.1833080

**Published:** 2026-06-26

**Authors:** Sumeya Mohamed, Muhammad Farooq Shaikh, Davide Piaggio

**Affiliations:** School of Engineering, University of Warwick, Coventry, United Kingdom

**Keywords:** accelerometry, epilepsy, focal seizures, systematic review and meta-analysis, Tonic–clonic seizures, wearable seizure detection

## Abstract

**Introduction:**

Wearable devices have emerged as promising tools for continuous seizure monitoring in patients with epilepsy. This study aimed to update the evidence on the diagnostic performance of non-invasive wearable sensors for seizure detection, extending the previous systematic review by Naganur et al., which included studies published up to November 10, 2021.

**Methods:**

A systematic review and meta-analysis were conducted using studies published up to July 15, 2025. Eligible studies used video-electroencephalographic (video-EEG) monitoring as the reference standard and reported sensitivity and false alarm rate (FAR) for wearable seizure detection devices. Included devices comprised wrist-worn and surface-worn sensors measuring parameters such as accelerometry, heart rate, and electrodermal activity.

**Results:**

Thirty-one studies evaluating tonic-clonic seizures (TCS) were included, comprising 2,128 patients and 112,542 h of video-EEG monitoring. The pooled sensitivity for TCS detection was 89.9% (95% CI: 85.0–93.9%), with a FAR of 1.43 per 24 h (95% CI: 1.22–1.64). Wrist-worn devices demonstrated slightly higher sensitivity (90.3%) but higher FAR (1.7/24 h) than surface devices (88.1% sensitivity; 0.8/24 h FAR). For focal seizures, nine studies involving 660 patients and 15,056 monitoring hours were included. The pooled sensitivity was 73.5% (95% CI: 57.4–87.0%) and the FAR was 2.85 per 24 h (95% CI: 1.40–5.81). Excluding one study that reported only overall sensitivity increased focal seizure sensitivity to 78.4%.

**Discussion:**

Wearable devices demonstrate high sensitivity for detecting tonic-clonic seizures, although false alarms remain a challenge. Detection performance for focal seizures is substantially lower, highlighting the need for improved algorithms and multimodal sensing approaches. These findings support the continued development of wearable technologies for real-world epilepsy monitoring and management.

## Introduction

Epilepsy is one of the most common chronic neurological disorders, affecting approximately 50 million people worldwide and imposing a substantial burden on health systems and society (Kwan and Brodie, 2000). Although many patients achieve good seizure control, approximately one-third have refractory epilepsy and continue to experience seizures despite appropriate antiepileptic drug (AED) therapy, which significantly impacts quality of life and safety (Kwan and Brodie, 2000; Löscher and Schmidt, 2011). Despite the introduction of newer AEDs over the past decades, evidence suggests that their efficacy has not substantially improved compared to older drugs, leaving a persistent population with drug-resistant epilepsy (Löscher and Schmidt, 2011). This unmet need has driven interest in complementary strategies such as seizure detection technologies.

Uncontrolled seizures carry serious risks, including sudden unexpected death in epilepsy (SUDEP). SUDEP is estimated to occur at rates ranging from 0.09 per 1,000 patient-years in newly diagnosed cases to as high as 9 per 1,000 in surgical candidates, and overall, sudden death is at least 20 times more common in people with epilepsy than in the general population (Hesdorffer et al., 2011; Nashef et al., 1995). Seizure detection devices are not only useful for alerting caregivers, reducing risks of SUDEP, and minimizing physical harm; they also play an important role in diagnosis and treatment. Currently, seizure diaries are widely used in both clinical practice and research, but they are known to be unreliable. A study found that patients failed to report 55.5% of all recorded seizures, including 73.2% of complex partial seizures and 85.8% of seizures that occurred during sleep (Elger and Hoppe, 2018). This under-reporting can lead to poor treatment decisions and inaccurate clinical trial outcomes.

Access to accurate seizure monitoring is especially limited in low-resource settings, where 80% of the global epilepsy burden exists. In these regions, even basic EEG access is scarce (Albarrak, 2024). Inpatient video-EEG monitoring (VEM), while considered the gold standard, is expensive, requires highly trained staff, and often involves long waiting periods (Vander et al., 2024). These challenges have led to growing interest in home-based and wearable seizure detection technologies that can offer more accessible and continuous monitoring (Naganur et al., 2022; Beniczky et al., 2021; Böttcher et al., 2022; Baumgartner et al., 2025).

The 2022 systematic review and meta-analysis by Naganur et al. quantified the performance of wearable devices in detecting tonic-clonic seizures (TCS), reporting high sensitivity (mean 91%) but relatively high false alarm rates (FARs) (Naganur et al., 2022). However, the review was limited to studies published up to November 2021 and did not include a meta-analysis of focal seizures or PNES due to insufficient data. Since then, several new studies have expanded the evidence base.

The present review updates and extends the work of Naganur et al. by incorporating newly published studies to the meta-analysis of TCS, in addition to conducting a meta-analysis of wearable device performance for detecting focal seizures, which was not feasible in the previous study (Naganur et al., 2022). This provides an updated benchmark for device accuracy and highlights ongoing gaps in the field.

## Methodology and materials

This systematic review and meta-analysis were conducted to update the work by Naganur et al., also following the search strategy by Beniczky et al. and PRISMA guidelines (Naganur et al., 2022; Beniczky et al., 2021; Page et al., 2021). As this study was conducted as an update and extension of a previously published systematic review, a separate PROSPERO registration was not completed prior to study initiation. A literature search was performed using the databases PubMed and Embase, with the following search string: ((automated detection) OR (algorithm AND detection) OR (wearable AND detection)) AND (epilepsy OR seizure). The search was limited to studies published after 15 November 2021, to update the original evidence base.

### Inclusion and exclusion criteria

Following Naganur et al. criteria, studies were included if they met all the following criteria:

- Population: Evaluated a wearable and non-invasive device in patients with epilepsy, or studies assessing differentiation between epileptic seizures and psychogenic non-epileptic seizures (PNES).
- Sample Size: Included a minimum of five participants.
- Measurement Modality: The primary measurement for seizure detection was not EEG.
- Outcomes Reported: Reported sensitivity for seizure detection and false alarm rate (FAR) as a measure over time.
- Validation Method: Devices and algorithms were trained and evaluated using non-invasive video-EEG monitoring as the gold standard comparator.

Studies were excluded if they:

- Evaluated nonwearable or invasive devices.
- Used wearable devices where EEG was primary modality.
- Did not report sensitivity or FAR.
- Were reviews, conference abstracts, or case reports with fewer than five participants.
- Lacked video-EEG validation.
- Focused on non-epileptic populations or seizure types not relevant to the scope of this review.

Although Naganur et al. stated that studies involving only paediatric patients were excluded, few such studies were still included in their descriptive and meta-analytic synthesis (Naganur et al., 2022). Therefore, in this update, this criterion was disregarded, to ensure comprehensive coverage of the available evidence and to address this inconsistency in prior methodology.

For each included study, the following data were extracted: first author, year of publication, device type, parameters measured, type of seizures, total number of participants, number of participants who experienced seizures, the number of VEM detected seizures, the number devices detected seizures, FAR/24, sensitivity, total recording time, and analysis method.

### Synthesis method

Two meta-analyses were conducted. This is the first updated analysis of tonic-clonic seizures (TCS) originally performed by Naganur et al. (2022) with the addition of the new eligible studies published since their review. Furthermore, this review also provides a new meta-analysis of focal seizures, which included the five (new) studies which were identified in their systematic review, but were not meta-analysed due to an insufficient number of studies at that time.

The quantitative synthesis was conducted using MetaXL version 5.3, a Microsoft Excel add-on for meta-analysis. For each included study, sensitivity (proportion of correctly detected seizures) and false alarm rate (FAR per 24 h) were extracted and meta-analysed separately (Elmakaty, 2025). As outcomes were expressed as proportions, pooled prevalence and rates methods were used. And the double arcsine transformation was applied to stabilize the variance, which is also the default method for prevalence (Elmakaty, 2025). MetaXL automatically back transformed the pooled estimates to proportions with corresponding 95% confidence intervals. Pooled estimates were generated using a random effect model, given the expected heterogeneity in device type, measurements modality, and patient populations. Heterogeneity was quantified using the Ι^2^statistic. Subgroup analysis was used to explore sources of heterogeneity, such as for device type and multimodal vs. unimodal systems. Results were presented as forest plots.

Moreover, MetaXL calculates study-level CIs using a normal approximation method, which are then displayed in the forest plot after applying the variance-stabilising transformation (Elmakaty, 2025). Meanwhile the original review conducted their analysis in Stata which appears to use the Clopper-Pearson method for calculating Cis (Elmakaty, 2025). This methodological difference explains why CIs might not match exactly between the two reviews. Publication bias was assessed using Doi plots.

### Quality assessment

The methodological quality and risk of bias of the included studies were assessed using predefined criteria adapted from the National Heart, Lung, and Blood Institute (NHLBI) quality assessment tool for observational and cross-sectional studies. These criteria were adapted to suit the assessment of wearable seizure detection studies and evaluated domains including study design, patient selection, reference standard quality, device methodology, and reporting of performance metrics such as sensitivity and false alarm rate (FAR). Each study was independently assessed by two reviewers, and any disagreements were resolved through discussion and consensus. The detailed quality assessment criteria and study-level evaluations are presented in Supplementary Table S1. In addition, the PRISMA 2020 checklist is provided in Supplementary Table S2.

## Results

**FIGURE 1:**
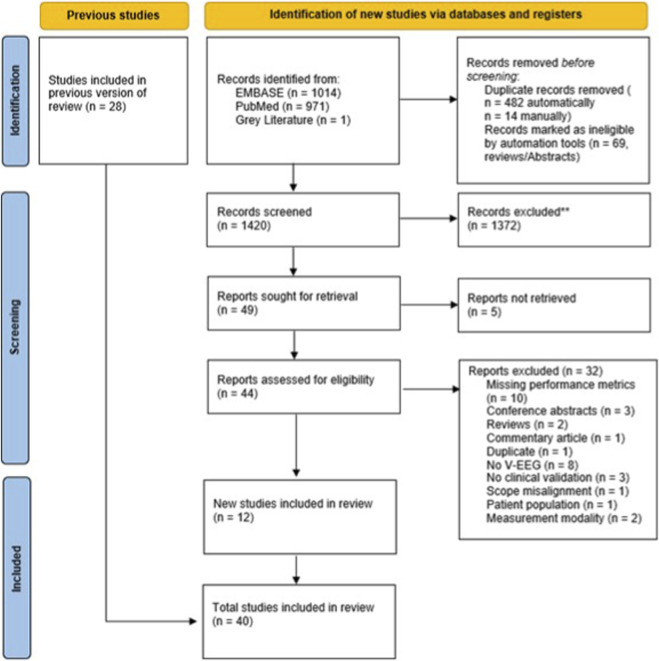
PRISMA flow chart for the updated meta-analysis and systematic review.

A total of 1,985 records were identified through the databases searched. After removal of 496 duplicates and 69 reviews or abstracts, 1,420 records were screened by title and abstract. Of these, 48 full-text articles were assessed for eligibility. Five full texts were not retrievable, either because the study itself or the manuscript was unavailable online. The study selection process is summarised in the PRISMA flow chart (Figure 1).

At the full-text stage, further exclusions were made for the following reasons: 9 studies did not report FAR and/or sensitivity; 6 were excluded for publication type (2 reviews, 3 conference abstract, 1 commentary); 8 lacked video-EEG validation, 3 had no clinical validation (i.e., relied only on public dataset); 2 studies were excluded as the detection system relied on EEG as a primary modality; 1 as a duplicate; 1 for scope misalignment; and 1 for patient population with neonatal seizures. This process resulted in 12 new studies that were included in the present update, of which 8 detected TCS seizure. The remaining 4 studies were used in the meta-analysis of focal seizures, of which 2 studies detected focal and TCS, 1 study detected focal impaired awareness seizures (FIAS), and one study detected focal onset motor seizures.

Although this review expanded the inclusion criteria to encompass paediatric populations, one study was excluded at full-text screening due to the population consisting solely of neonates (Chen et al., 2023). According to the ILAE classification, most neonatal seizures are acute symptomatic and do not meet the diagnostic criteria for epilepsy unless they are part of a defined epilepsy syndrome (Pressler et al., 2021). Therefore, the study was excluded based on population not meeting the epilepsy or PNES criteria.

### Meta-analysis of TCS

A total of 31 studies were included in the meta-analysis of tonic-clonic seizures (TCS), of which 23 studies come from the previous review, while 8 studies come from the present update (Naganur et al., 2022). Among these 8 additional studies, one study focused on detecting focal to bilateral tonic-clonic seizures (FBTCS), and another focused on detecting tonic seizures (TS). A summary of the extracted data for all 31 studies is presented in Table 1.

**TABLE 1:** Summary of studies detecting TCS.

First author, year	Device type	Parameters measured	Patients who had seizures	Total patients recruited	VEM: seizures	Device: seizures	False alarm rate, per 24 h	Sensitivity
Beniczky et al. (2013)	Wrist-worn	3D accelerometry	20	73	39	35	0.2	89.7%
Beniczky et al. (2018)	Wearable surface device	sEMG signals	20	71	32	30	0.67	93.8%
Conradsen et al. (2012)	Wearable surface device	sEMG signals	2	5	7	4	0.07	57.1%
Conradsen et al. (2011)	Wearable surface device	One sEMG signal	11	60	22	22	1	100%
De Cooman et al. (2018)	Wrist-worn and wearable surface device	3D accelerometry, heart rate, sEMG	7	7	22	21	16.8	95.5%
Halford et al. (2017)	Wearable surface device	sEMG signals	24	149	29	29	1.44	100%
Johansso et al. (2019)	Wrist-worn	3D accelerometry	856	75	10	10	1.2	100%
Kramer et al. (2011)	Wrist-worn	3D accelerometry	15	31	22	20	0.11	90.9%
Kusmakar et al. (2017)	Wrist-worn	3D accelerometry	12	12	21	20	0.72	95.2%
Kusmakar et al. (2018a)	Wrist-worn	3D accelerometry	11	16	8	6	0.59	75.0%
Kusmakar et al. (2018b)	Wrist-worn	3D accelerometry	8	8	9	9	1.1	100%
Kusmakar et al. (2018c)	Wrist-worn	3D accelerometry	14	79	26	25	0.64	96.2%
Kusmakar et al. (2016)	Wrist-worn	3D accelerometry	11	16	21	21	0.73	100%
Larsen et al. (2014)	Wearable surface device	sEMG signals	6	6	26	14	53.2	55.8%
Milošević et al. (2014)	Wrist- and ankle-worn	3D accelerometry, heart rate, sEMG	14	56	117	117	9.36	100%
Milošević et al. (2015)	Wrist-worn	3D accelerometry, sEMG	7	56	22	20	1	91.0%
Naganur et al. (2019)	Wrist-worn	3D accelerometry	5	26	23	11	2.43	47.8%
Onorati et al. (2017)	Wrist-worn	EDA, 3D accelerometry	22	69	55	52	0.19	94.5%
Onorati et al. (2021)	Wrist-worn and wearable surface device	EDA, 3D accelerometry	18	85	35	32	1.26	92.0%
Onorati et al. (2021)	Wrist-worn and wearable surface device	EDA, 3D accelerometry	18	67	31	29	0.57	94.0%
Poh et al. (2012)	Wrist-worn	EDA, 3D accelerometry	7	80	16	15	0.74	93.8%
Szabó et al. (2015)	Wearable surface device	sEMG signals	11	33	21	20	0.02	95.2%
Tang et al. (2021)	Wrist-worn	EDA, 3D accelerometry	94	94	548	438	13.6	80.0%
van Andel et al. (2017)	Wrist-worn	3D accelerometry, heart rate	23	95	86	61	6.9	71.0%
Larsen et al. (2024)	Wrist-worn	3D Accelerometry, 3D Gyroscope	15	33	70	70	0.32	100%
Spahr et al. (2025)	Wrist-worn	3D Accelerometry	33	384	49	47	0.13	96.0%
Böttcher et al. (2021)	Wrist-worn	EDA, 3D Accelerometry PPG	10	243	11	10	0.19	91.0%
Agrahri et al. (2022)	Wrist-worn	3D Accelerometry	35	79	46	44	1.94	95.7%
Gharbi et al. (2024)	Connected Shirt	3D accelerometry, heart rate	42	42	66	56	0.55	84.8%
Halimeh et al. (2023)	Wrist-worn/Ankle-worn	EDA, 3D accelerometry, heart rate	23	76	45	23	0.28	52.0%
Vakilna et al. (2024)	wrist-worn	3D Accelerometry, 3D Gyroscope, PPG	11	36	23	22	0.21	87.0%

Most studies employed wrist-worn devices (n = 20), while 2 studies assessed wrist/ankle-worn devices. 8 studies utilised a wearable surface device, 1 used a connected shirt, and 2 studies used a wearable surface device as well as a wrist-worn device. Across these studies,

15 were based on a multi-modal system, while 16 employed a unimodal device. The parameters measured included 3D accelerometry (ACC), surface electromyography (sEMG), heart rate (HR), electrodermal activity (EDA), gyroscopy (GYRO), and photoplethysmography (PPG).

The 31 studies resulted in a total of 112,542.2 h of VEM recording time across a total of 2,128 recruited patients, corresponding to an average of 52.9 h per patient. This average remains unchanged from the previous review (Naganur et al., 2022). Of these, 567 patients had seizures during monitoring, with a total of 1,618 seizures recorded.

**FIGURE 2:**
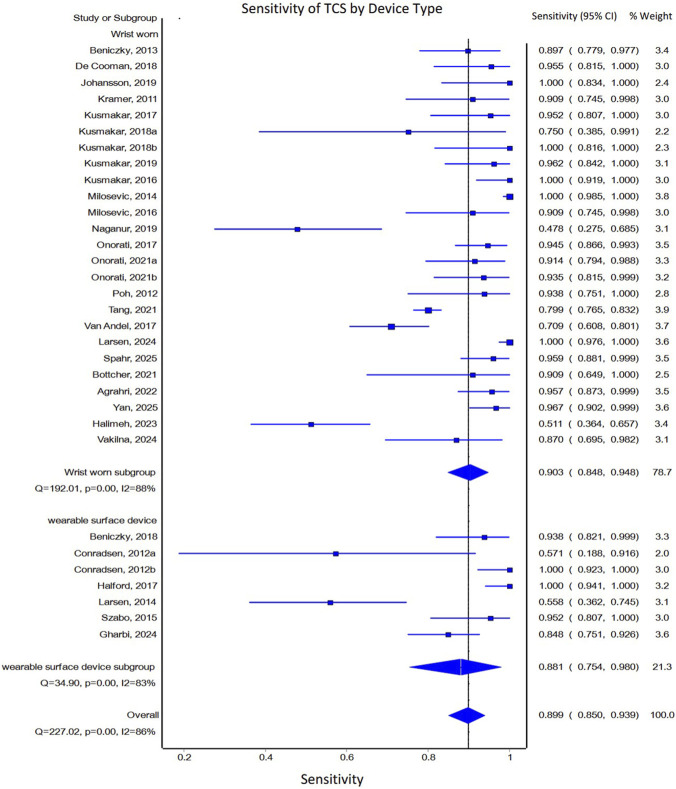
Forest plot of sensitivity for detection of TCS across 31 studies. Individual study estimates are shown with 95% confidence intervals, with pooled estimates calculated using a random-effects model with double arcsine transformation. Subgroup analyses were performed by device type (wrist-worn vs. wearable surface device).

**FIGURE 3:**
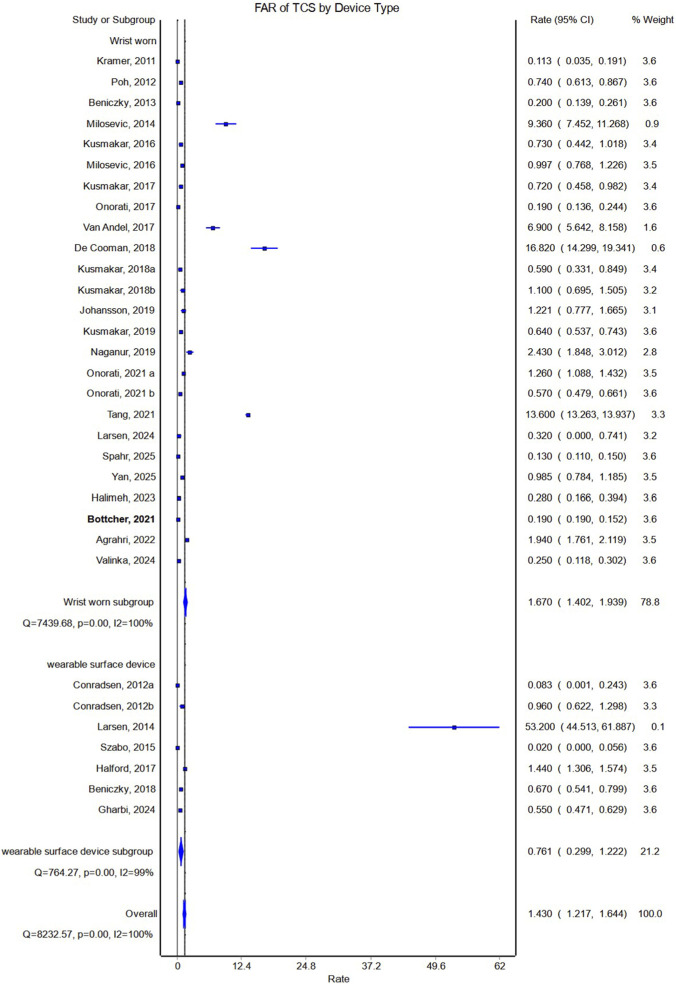
Forest plot of false alarm rate (FAR, per 24 h) for detection of TCS across 31 studies. Individual study estimates are shown with 95% CIs, with pooled estimates. Subgroup analyses were performed by device type (wrist-worn vs. wearable surface device).

As shown in Figure 2, the pooled sensitivity across studies detecting TCS was 89.9% (95% CI: 85.0%–93.9%). Heterogeneity between studies was high with Ι^2^ = 86.3% (95% CI: 81.8%–89.8%). Subgroup analysis based on device type, shows a pooled sensitivity of 90.3% (95% CI: 84.8%–94.8%, Ι^2^ = 88%) for wrist-worn devices and 88.1% (95% CI: 75.4%–98.0%, Ι^2^ = 83%) for surface wearable devices. The pooled FAR across the 31 studies was 1.43/2 4 h [95% CI: 1.22/24 h–1.64/24 h], with similarly high heterogeneity Ι^2^ = 99.6% (95% CI: 99.6%–99.7%) (Figure 3). FAR for the wrist-worn subgroup was 1.7/24 h (95% CI: 1.4/24 h–1.9/24 h%, Ι^2^ = 100%), while FAR for the wearable surface device was 0.8/24 h (95% CI: 0.3/24 h–1.2/24 h%, Ι^2^ = 99%). Doi plots showed minor asymmetry for sensitivity (LFK index = 1.13), whereas FAR analyses showed major asymmetry (LFK index = 5.3).

Therefore, pooled FAR estimates should be interpreted cautiously, as substantial heterogeneity likely reflects important methodological and clinical differences between included studies rather than a single underlying effect size.

### Meta-analysis of focal seizures

A total of 9 studies were included in the meta-analysis evaluating the performance of wearable devices detecting focal seizures, with 5 studies obtained from the previous review and 4 newly identified in the current search. Of these, 5 studies detected both TCS and focal seizures, reporting seizure-type-specific sensitivity. Three studies focused only on focal seizures. One study detected both TCS and focal seizures but did not report seizure-type-specific sensitivity; however, it was included in the pooled sensitivity analysis as the dataset was predominantly focal seizures (40 out of 51). Table 2 summarises the characteristics of these studies. Seven studies utilised a wearable surface device measuring heart rate and heart rate variability (HRV). One study utilised a wrist-worn device that measured accelerometry (ACC), electrodermal activity (EDA), and blood volume pulse (BVP). One study used a biometric shirt with sensors for accelerometry, electrocardiography (ECG), and respiratory signals.

**TABLE 2:** Summary of 4 studies detecting Focal seizures, and 5 studies detecting TCS and Focal seizures.

First author, year published	Device type	Parameters measured	Type of seizure	Patients who had seizures	Total patients recruited	VEM: seizures	Device: seizures	False alarm rate, per 24 h	Sensitivity
Jeppesen et al. (2019)	Wearable surface device	HR and HRV	TCS and focal seizures	23	100	18 TCS, 108 focal	17 TCS, 97 focal	1	Overall: 93.10% Focal: 89.8% TCS: 94.4%
Jeppesen et al. (2020)	Wearable surface device	HR and HRV	TCS and focal seizures	11	19	10 TCS, 12 focal	9 TCS, 10 focal	0.9	Overall: 87% Focal: 83.3% TCS: 90%
Hegarty-Craver et al. (2021)	Wearable surface device	HR and HRV	TCS and focal seizures	25	40	12 TCS, 13 focal	11 TCS, 7 focal	1.03	Overall: 72% Focal: 53.8% TCS: 91.7%
Jahanbekam et al. (2021)	Wearable surface device	HR and HRV	TCS and focal seizures	30	30	51	16	1.2	31.1%
Vandecasteele et al. (2017)	Wearable surface device	HR and HRV	Focal seizures	11	11	47	33	50.6	70%
St-Jean et al. (2025)	Biometric Shirt	Respiratory, 3D accelerometry, HR	FIAS	27	27	113	71	30	66%
Jeppesen et al. (2024)	wearable surface device	HR and HRV	TCS and focal seizures	22	39	26 TCS,33 focal	25 TCS, 25 focal	0.25	overall: 84.8%, Focal:75.8%, TCS: 96.2%
Bottcher et al. (2022)	Wrist-worn	3D Accelerometry, EDA, BVP	Focal onset motor seizures	9	243	20	20	13.4	75%
Jeppeson et al. (2023)	wearable surface device	HR and HRV	TCS and focal seizures	11	19	10 TCS, 13 focal	8 TCS, 10 focal	0.62	overall: 78.2%, Focal: 76.9%, TCS: 80%

A total recording time of 15,056.5 h was reported across 660 recruited patients, giving an average VEM recording time of 22.8 h per patient. In these 9 studies, a total of 463 seizures were recorded during VEM, of which 386 (83.4%) were focal seizures, 70 (15.1%) were TCS, and 7 (1.5%) were unclassified.

**FIGURE 4:**
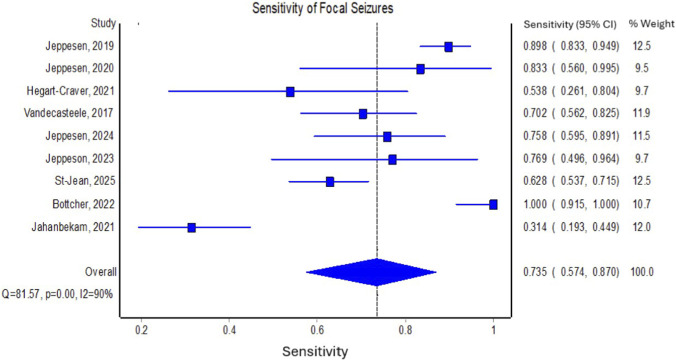
Forest plot of sensitivity for detection of focal seizures across 9 studies. Individual study estimates are shown with 95% Cls, with pooled estimates calculated using a random-effects model with double arcsine transformation.

**FIGURE 5:**
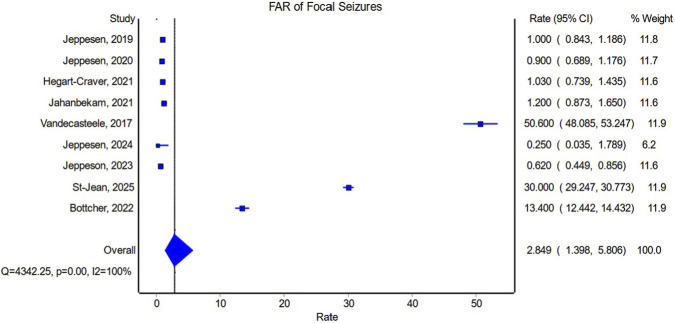
Forest plot of FAR (per 24 h) for detection of focal seizures across 9 studies. Individual study estimates are shown with 95% Cls.

As shown in Figure 4, the pooled sensitivity across the 9 studies was 73.5% (95% CI: 57.4%–87.0%), with high heterogeneity, Ι2 = 90.2% (95% CI: 83.6%–94.1%). The pooled FAR was 2.85/24 h (95% CI: 1.40/24 h–5.81/24 h), similarly with high heterogeneity, Ι2 = 99.82% (95% CI: 99.879%–99.84%), Figure 5. A sensitivity analysis excluding the study that did not report seizure-type-specific sensitivity resulted in a pooled sensitivity of 78.4% (95% CI: 65.8%–88.8%, Ι2 = 82.9%).

Doi plots for sensitivity of focal seizures showed no asymmetry with LFK index of 0.85. As for FAR the plot showed major asymmetry with LFK index of −10.3.

No newly identified studies focused primarily on PNES detection, and therefore no PNES-specific quantitative synthesis was performed in the present update.

## Discussion

The current systematic review and meta-analysis build on the findings of Naganur et al. (2022) and offers a timely summary of the effectiveness of non-invasive wearable seizure detection devices. The pooled estimates for tonic–clonic seizure (TCS) detection are based on a larger evidence base, with 12 more studies being included. To the best of our knowledge, this review also provides the first meta-analysis of focal seizure detection using wearable devices, addressing a key limitation of the earlier review (Naganur et al., 2022).

The combined sensitivity of TCS detection remained high at 89.9%. Re-analysis of the original evidence base in MetaXL yielded the same estimate, and sensitivity was similar to what was reported by Naganur et al. (2022): 91.1%. Pooled false alarm rate (FAR) decreased to 1.43/24 h from the previous estimate of 2.1/24 h, but wrist-worn devices still had higher FARs than the surface wearable devices. In the eight new studies added to TCS, all resulted in values below 2/24 h and seven of the new studies reported values below 1/24 h. Focal seizure detection, on the other hand, had a lower pooled sensitivity of 73.5% and higher FAR of 2.9/24 h. Three studies tested only focal seizures; they reported sensitivities of 66%, 70%, and 75%. These studies also reported the highest FARs, at 30/24 h, 50.6/24 h, and 13.4/24 h, respectively. This study is in line with the current ILAE recommendations which state that the use of wearable detection is more restricted in seizure types with less conspicuous body movements such as convulsions (Beniczky et al., 2021).

Accelerometry is currently the most robust and consistently applied sensing modality for the wearable seizure detection devices (Beniczky et al., 2013; Johansso et al., 2019; Kusmakar et al., 2017; Kusmakar et al., 2018a; Kusmakar et al., 2018b; Kusmakar et al., 2018c; Kusmakar et al., 2016; Milošević et al., 2014; Milošević et al., 2015; Poh et al., 2012; Tang et al., 2021). Compared to motion-based sensors (Naganur et al., 2022; Onorati et al., 2017; Poh et al., 2012; Böttcher et al., 2021), cardiac-based modalities have a lesser history of being used for the detection of convulsive seizures. But there is a new evidence base indicating an increased interest in using a combination of autonomic signals and motion-based features to enhance the detection performance (Onorati et al., 2017; Onorati et al., 2021; Poh et al., 2012; Böttcher et al., 2021). Of note, two studies employed photoplethysmography (PPG) instead of electrocardiography (ECG) for extracting the HR, but Böttcher et al. found that ictal movement artefacts (Böttcher et al., 2021; Vakilna et al., 2024) had significant impacts on the photoplethysmographic signals.

As in the last review, most of the seizure detection methods used were based on cardiac parameters such as heart rate and heart-rate variability (Jeppesen et al., 2019; Jeppesen et al., 2020; Hegarty-Craver et al., 2021; Jahanbekam et al., 2021; Jeppesen et al., 2024). It is known that ictal tachycardia is a physiological marker of seizures, and it has been extensively studied for the purposes of seizure detection (Vakilna et al., 2024; Jeppesen et al., 2019; Jeppesen et al., 2020; Hegarty-Craver et al., 2021; Jahanbekam et al., 2021). But not all individuals have ictal tachycardia. The incidence of ictal tachycardia was 82% in a review of 34 studies, with threshold ranging from more than 10 bpm above baseline to an absolute threshold of over 100 or 120 bpm, and 71% of all focal seizures (Eggleston et al., 2014). This variability is supportive of the idea of “responders” and “non-responders” to seizure detection based on cardiac parameters.

The Jeppesen series of studies included patients with an increase in heart rate greater than 50 bpm during seizures. The highest detection sensitivity was consistently achieved by responders (up to 93.1%) and the lowest FARs were achieved (as low as 0.25/24 h). However, those who did not respond did worse, indicating that pre-screening or patient-specific modelling would be required to achieve better results and that there may be a tendency to select responder patients. Patient-adaptive HRV thresholds in this series were better than predefined thresholds in terms of reducing FAR with maintaining sensitivity (Jeppesen et al., 2019; Jeppesen et al., 2020; Jeppesen et al., 2024; Jeppesen et al., 2023). Likewise, St-Jean et al. did not pre-select patients, but this exclusion of non-responders increased the sensitivity of the test from 66% to 81%, reinforcing the importance of adaptive modelling or responder-based stratification (St‐Jean et al., 2025).

There is some degree of potential, although heart rate-based detection methods may suffer from poor specificity as the heart rate might also be affected by non-seizure physiological and behavioural factors (Baumgartner et al., 2025; Jeppesen et al., 2024; St‐Jean et al., 2025). Several studies have thus examined multimodal approaches that integrate cardiac signals with motion signals and/or other biosignals for improving the performance of seizure detection (Onorati et al., 2017; Van Andel et al., 2017; Böttcher et al., 2021; Wang et al., 2025). Jahanbekam et al. did not find any benefit of the inclusion of accelerometry and electrodermal activity alongside ECG, while Hegarty-Craver et al. reported that including motion data did improve the detection latency, but not the overall sensitivity. Vandecasteele et al. demonstrated the better performance of wearable ECG than PPG in detecting seizures, likely due to the insensitivity of the former to motion artefacts (Hegarty-Craver et al., 2021; Jahanbekam et al., 2021; Jeppesen et al., 2023; Vandecasteele et al., 2017). The results suggest that cardiac-based methods continue to play a key role in detecting focal seizures, and future advancements will rely on the development of personalized models, effective signal processing techniques, and well-designed multimodal integration methods.

As highlighted by Naganur et al., one major methodological limitation in wearable seizure detection research is the potential overlap between training and testing datasets, which may inflate reported performance estimates (Naganur et al., 2022; Naganur et al., 2019; Tang et al., 2021). This was addressed in several studies in the updated evidence base by using prospective validation frameworks and independent testing sets (Spahr et al., 2025; Böttcher et al., 2021; Halimeh et al., 2023; Eggleston et al., 2014). Vakilna et al. performed a fixed-and-frozen prospective validation study in which a pre-trained algorithm was tested on an independent patient cohort without re-training or adjustment of the parameters, which is more realistic for out-of-sample performance (Vakilna et al., 2024). Other investigations also employed independent test sets which is a very significant methodological advance, as it minimizes bias and enhances generalisability to real world situations (Spahr et al., 2025; Böttcher et al., 2021; Gharbi et al., 2024; Wang et al., 2025).

### Sources of heterogeneity and implications for future research

The different sources of heterogeneity are identified and implications for research are discussed.

One of the key findings of this review was the significant degree of heterogeneity that was seen in the sensitivity and FAR analyses, especially in the FAR analyses. This variability was probably due to several methodological and clinical factors. First, there were significant differences among the types of seizures included in the studies (Larsen et al., 2024; Vakilna et al., 2024; St‐Jean et al., 2025); some included generalized tonic–clonic; some included focal impaired awareness seizures; and some included focal onset motor seizures. The motor and physiological manifestations of these seizure types are quite different and directly affect the performance of the wearables for detecting seizures (Beniczky et al., 2021).

Second, there was considerable variation in the modalities and the configuration of the devices. Studies included a variety of combinations of accelerometers, EDA, HR, HRV, sEMG, PPG, respiratory monitoring and multimodal sensor fusion (Beniczky et al., 2018; Onorati et al., 2017; Poh et al., 2012; Böttcher et al., 2021; St‐Jean et al., 2025; Wang et al., 2025). Inter-study variations may have been significant due to differences in signal quality, sensor placement, preprocessing techniques, and feature extraction algorithms.

Thirdly, methodological heterogeneity occurred in study design and validation strategies. Some studies used datasets that were collected in the past or cohorts that were partially overlapping between the training and testing sets (Naganur et al., 2022; Spahr et al., 2025; Böttcher et al., 2021; Vakilna et al., 2024), while others performed prospective validation and external independent testing. Direct comparisons between the studies were further complicated by variability in the machine learning methods, definitions of thresholds, patient-specific adaptation methods and reporting standards for performance.

Heterogeneity may also have been partly attributable to population differences. Others studied highly selected populations of responders, or only patients who had seizures during video-EEG monitoring (Jeppesen et al., 2019; Jeppesen et al., 2020; Jeppesen et al., 2024; Jeppesen et al., 2023). Additionally, substantial variability existed in sensor type, placement, and algorithmic design across studies. Included systems ranged from wrist-worn and ankle-worn devices to wearable surface patches and biometric shirts, each with differing susceptibility to motion artefacts and signal noise (Beniczky et al., 2018; Böttcher et al., 2021; Gharbi et al., 2024; Vakilna et al., 2024; St‐Jean et al., 2025; Wang et al., 2025). Furthermore, some studies relied on predefined threshold-based approaches, whereas others utilised machine-learning or patient-adaptive models, introducing additional variability in detection performance and FAR outcomes (Spahr et al., 2025; Vakilna et al., 2024; Jeppesen et al., 2024; Jeppesen et al., 2023; Wang et al., 2025).

The high level of heterogeneity in the FAR analyses could also be due to variations in definitions and reporting of false alarms. Some studies reported FAR for all monitoring periods, while others omitted artefactual recordings, sleep periods or non-wear time (Naganur et al., 2022; Böttcher et al., 2021; Vakilna et al., 2024). This may have affected estimates of pooled FAR because of differences in monitoring time and in units of reporting.

The results highlight the importance of future research to be more methodologically standardised in the field of wearable seizure detection. Prospective, multicentre validation, independent external test cohorts, a standardised seizure classification, harmonised reporting of sensitivity and FAR metrics, and reporting of preprocessing and machine-learning methodologies should be prioritised in future studies (Naganur et al., 2022; Beniczky et al., 2021). To enhance the diversity of datasets and representation of under-represented seizure types, including focal seizures and PNES-related differential diagnosis studies, will further strengthen the robustness and generalisability of wearable seizure detection devices.

In addition, several studies reported substantially elevated FAR values, particularly in focal seizure detection cohorts, which may have disproportionately influenced the pooled FAR estimates and contributed to the observed heterogeneity. These extreme FAR values may reflect differences in detection thresholds, susceptibility to motion artefacts, small seizure-positive cohorts, patient-specific variability, or limitations associated with certain sensing modalities. Consequently, pooled FAR estimates should be interpreted cautiously, particularly given the substantial methodological and clinical variability across included studies.

## Limitation

Several limitations persist across the available literature and should be addressed in future studies. Many studies continue to rely on epilepsy monitoring unit and video-EEG environments that may not fully reflect real-world ambulatory performance. Several studies involved relatively small and selective patient cohorts, often restricted to individuals who experienced seizures during monitoring, which may contribute to selection bias and overestimation of sensitivity. However, the possibility of partially overlapping patient cohorts between studies cannot be completely excluded, particularly among studies originating from the same research groups or institutions. Although efforts were made to identify and minimise potential duplication during study selection and data extraction, incomplete reporting of cohort characteristics in some studies limited definitive verification.

Many included studies were additionally limited by relatively small sample sizes and low numbers of recorded seizures, particularly within focal seizure cohorts. Several studies also restricted analyses to patients who experienced seizures during monitoring, which may have contributed to inflated performance estimates and reduced generalisability of the reported findings. Most included studies were conducted in controlled inpatient video-EEG monitoring environments, which may not fully reflect the performance of wearable seizure detection systems in real-world ambulatory settings. Factors such as unrestricted patient movement, environmental noise, inconsistent device adherence, and reduced supervision in ambulatory environments may influence detection performance and false alarm rates differently from inpatient VEM conditions.

Furthermore, this update confirms the limited evidence available for psychogenic non-epileptic seizures (PNES), as no additional PNES-focused studies were identified beyond the original review. Although PNES are not classified as epileptic seizures according to ILAE definitions, PNES-related studies were retained for methodological consistency with the original review by Naganur et al. and because several wearable detection studies specifically investigated differentiation between epileptic seizures and PNES (Naganur et al., 2022; Kusmakar et al., 2016; Naganur et al., 2019). Importantly, no PNES events were included in the pooled meta-analytic estimates reported in the present review, and the focal seizure analyses were restricted to epileptic seizure outcomes only. While paediatric studies were incorporated to improve comprehensiveness, neonatal seizure studies were excluded because neonatal seizures do not typically meet the diagnostic criteria for epilepsy unless they occur as part of a defined epilepsy syndrome.

An additional limitation of this review is the restricted database search strategy, which was limited to PubMed and Embase. Although these databases capture a substantial proportion of clinically relevant epilepsy and biomedical literature, it is possible that engineering-focused studies indexed primarily in databases such as IEEE Xplore, Scopus, or Web of Science were not identified. However, the search strategy was intentionally aligned with the methodology of the original review to maintain methodological consistency and comparability between evidence updates, while prioritising studies with clinically validated seizure detection systems using video-EEG as the reference standard. Furthermore, this review was not prospectively registered in PROSPERO prior to study initiation. Although predefined eligibility criteria and PRISMA-guided methodology were followed throughout the review process, the absence of prospective registration may limit methodological transparency and should therefore be considered when interpreting the findings.

## Conclusion

The findings of this updated review highlight some improvements in tonic-clonic seizure detection, particularly in terms of the reduction of FAR, while sensitivity remains unchanged but acceptable. Furthermore, future studies will continue to maintain a balance between high sensitivity and reduced FAR, the clinical suitability of wearable devices for TCS detection and monitoring in clinical and potentially ambulatory settings will continue to grow. However, the performance gap for focal seizures remains a challenge, as seen by the pooled sensitivity and FAR. Despite the promising findings for focal seizure detection, the current evidence base remains relatively limited compared with tonic–clonic seizure detection studies. Therefore, the pooled estimates for focal seizures should be interpreted cautiously, particularly given the relatively small number of studies, limited seizure-positive cohorts, and substantial inter-study heterogeneity.

This reflects the need for further research into sensing modalities that can capture subtle physiological or neural signals, and into approaches that integrate and complement existing methods. Future research should focus on expanding datasets to include underrepresented seizure types especially PNES and focal seizures without prominent motor features. Methodological rigor must also continue to improve, especially through prospective validations and independent testing to ensure generalisability, as well as standardising the reporting of performance metrics as it would allow for easier comparison between systems. These steps will help move the field of wearables for seizure detection forward.

## Data Availability

The datasets presented in this study can be found in online repositories. The names of the repository/repositories and accession number(s) can be found in the article/Supplementary Material.
