# Supplementary material for: Automated seizure detection using wearable devices: updated systematic review and meta-analysis of tonic-clonic and focal seizure detection
Source: Front Bioeng Biotechnol. 2026 Jun 26;14:1833080. doi: 10.3389/fbioe.2026.1833080 (PMC13350324; doi:10.3389/fbioe.2026.1833080)
Supplement: Supplementary file 1 [file Table1.DOCX]

**Table S1; Supplementary Data: The assessment of risk of bias using the National Heart, Lung, and Blood institute (NHLBI) tool.**

| **Criteria** | **Was the research question or objective in this paper clearly stated?** | **Was the study population clearly specified and defined?** | **Was the participation rate of eligible persons at least 50%?** | **Were all the subjects selected or recruited from the same or similar populations (including the same time period)? Were inclusion and exclusion criteria for being in the study prespecified and applied uniformly to all participants?** | **Was a sample size justification, power description, or variance and effect estimates provided?** | **For the analyses in this paper, were the exposure(s) of interest measured prior to the outcome(s) being measured?** | **Was the timeframe sufficient so that one could reasonably expect to see an association between exposure and outcome if it existed?** | **For exposures that can vary in amount or level, did the study examine different levels of the exposure as related to the outcome (e.g., categories of exposure, or exposure measured as continuous variable)?** | **Were the exposure measures (independent variables) clearly defined, valid, reliable, and implemented consistently across all study participants?** | **Was the exposure(s) assessed more than once over time?** | **Were the outcome measures (dependent variables) clearly defined, valid, reliable, and implemented consistently across all study participants?** | **Were the outcome assessors blinded to the exposure status of participants?** | **Was loss to follow-up after baseline 20% or less?** | **Were key potential confounding variables measured and adjusted statistically for their impact on the relationship between exposure(s) and outcome(s)?** | **Good, Fair, Poor** |
| --- | --- | --- | --- | --- | --- | --- | --- | --- | --- | --- | --- | --- | --- | --- | --- |
| **Kusmakar et al. 2018** | Yes | Yes | Yes –reported as consecutive series | Yes | No | No | Yes | N/A | Yes | N/A | Yes | No | Yes | No | Fair |
| **Kusmakar et al. 2019** | Yes | Yes | Not reported | Yes | No | No | Yes | N/A | Yes | N/A | Yes | Yes | Yes | No | Fair |
| **Beniczky et al. 2018** | Yes | Yes | Yes | Yes | Yes | No | Yes | N/A | Yes | N/A | Yes | Yes | Yes | No | Good |
| **Halford et al. 2017** | Yes | Yes | Not reported | Yes | No | No | Yes | N/A | Yes | N/A | Yes | Yes | No | Yes | Good |
| **Kusmakar et al. 2016** | Yes | Yes | Not reported | Yes | No | No | Yes | N/A | Yes | N/A | Yes | No | Yes | No | Fair |
| **Larsen et al. 2014** | Yes | Yes | Not reported | Yes | No | No | Yes | N/A | Yes | N/A | Yes | No | Yes | No | Fair |
| **Beniczky et al. 2013** | Yes | Yes | Yes –reported as consecutive series | Yes | No | No | Yes | N/A | Yes | N/A | Yes | Yes | Yes | No | Good |
| **Conradsen et al. 2012** | Yes | Yes | Yes –reported as consecutive series | Yes | No | No | Yes | N/A | Yes | N/A | Yes | Yes | Yes | No | Fair |
| **Conradsen et al. 2012** | Yes | Yes | Yes –reported as consecutive series | Yes | No | No | Yes | N/A | Yes | N/A | Yes | No | Yes | No | Fair |
| **Naganur et al. 2019** | Yes | Yes | Not reported | Yes | No | No | Yes | N/A | Yes | N/A | Yes | Yes | Yes | No | Fair |
| **Onorati et al. 2017** | Yes | Yes | Not reported | Yes | No | Yes | Yes | N/A | Yes | N/A | Yes | No | Yes | No | Good |
| **Poh et al. 2012** | Yes | Yes | Not reported | Yes | No | Yes | Yes | N/A | Yes | N/A | Yes | No | Yes | No | Good |
| **Kramer et al. 2011** | Yes | Yes | Yes –reported as consecutive series | Yes | No | No | Yes | N/A | Yes | N/A | Yes | No | Not reported | No | Fair |
| **Kusmakar et al. 2017** | Yes | Yes | Not reported | Yes | No | Yes | Yes | N/A | Yes | N/A | Yes | No | Yes | No | Fair |
| **Kusmakar et al. 2018** | Yes | Yes | Not reported | Yes | No | Yes | Yes | N/A | Yes | N/A | Yes | No | Yes | No | Fair |
| **Szabo et al. 2015** | Yes | Yes | Yes –reported as consecutive series | Yes | No | No | Yes | N/A | Yes | N/A | Yes | No | Yes | No | Fair |
| **Van Andel et al. 2017** | Yes | Yes | Not reported | Yes | No | Yes | Yes | N/A | Yes | N/A | Yes | Yes | No | No | Fair |
| **Johannson et al. 2019** | Yes | Yes | Not reported | Yes | No | Yes | Yes | N/A | Yes | N/A | Yes | Yes | No | No | Fair |
| **Milosevic et al. 2014** | Yes | Yes | Not reported | Yes | No | No | Yes | N/A | Yes | N/A | Yes | No | Yes | No | Fair |
| **De Cooman et al. 2018** | Yes | Yes | Not reported | Yes | No | No | Yes | N/A | Yes | N/A | Yes | No | Yes | No | Fair |
| **Milosevic et al. 2016** | Yes | Yes | Not reported | Yes | No | No | Yes | N/A | Yes | N/A | Yes | No | Yes | No | Fair |
| **Onorati et al. 2021** | Yes | Yes | Yes | Yes | Yes | Yes | Yes | N/A | Yes | N/A | Yes | Yes | Yes | No | Good |
| **Tang et al. 2021** | Yes | Yes | Not reported | Yes | No | No | Yes | N/A | Yes | N/A | Yes | No | Yes | No | Fair |
| **Jeppesen et al. 2019** | Yes | Yes | Yes –reported as consecutive series | Yes | No | No | Yes | N/A | Yes | N/A | Yes | Yes | Yes | No | Good |
| **Jeppesen et al. 2020** | Yes | Yes | Yes –reported as consecutive series | Yes | No | No | Yes | N/A | Yes | N/A | Yes | Yes | Yes | No | Good |
| **Hegarty-Craver et al. 2021** | Yes | Yes | Not reported | Yes | No | Yes | Yes | N/A | Yes | N/A | Yes | No | Yes | No | Fair |
| **Jahanbekam et al. 2021** | Yes | Yes | Not reported | Yes | No | Yes | Yes | N/A | Yes | N/A | Yes | No | Yes | No | Fair |
| **Vandecasteele et al. 2017** | Yes | Yes | Not reported | Yes | No | Yes | Yes | N/A | Yes | N/A | Yes | No | Yes | No | Fair |
| **Larsen et al, 2024** | Yes | Yes | Not reported | Yes | No | No | Yes | N/A | Yes | N/A | Yes | Yes | No | No | Fair |
| **Spahr et al, 2025** | Yes | Yes | Not reported | Yes | No | No | Yes | N/A | Yes | N/A | Yes | Yes | No | Yes | Good |
| **Bottcher et al, 2021** | Yes | Yes | Not reported | Yes | No | No | Yes | N/A | Yes | N/A | Yes | No | Yes | No | Fair |
| **Agrahri et al, 2022** | Yes | Yes | Not reported | Yes | No | No | Yes | N/A | Yes | N/A | Yes | Yes | Yes | No | Fair |
| **Yan et al, 2025** | Yes | Yes | Not reported | Yes | No | No | Yes | N/A | Yes | N/A | Yes | Yes | No | No | Fair |
| **Gharbi et al, 2024** | Yes | Yes | Not reported | Yes | No | No | Yes | N/A | Yes | N/A | Yes | Yes | Yes | No | Good |
| **Halimeh et al, 2023** | Yes | Yes | Not reported | Yes | No | No | Yes | N/A | Yes | N/A | Yes | No | Yes | No | Fair |
| **Vakilna et al, 2024** | Yes | Yes | Not reported | Yes | No | No | Yes | N/A | Yes | N/A | Yes | Yes | Yes | No | Good |
| **St-Jean et al, 2025** | Yes | Yes | Not reported | Yes | No | No | Yes | N/A | Yes | N/A | Yes | Yes | Yes | No | Good |
| **Jeppesen et al, 2024** | Yes | Yes | Not reported | Yes | No | No | Yes | N/A | Yes | N/A | Yes | Yes | Yes | No | Good |
| **Bottcher et al, 2022** | Yes | Yes | No | Yes | No | No | Yes | N/A | Yes | N/A | Yes | No | No | No | Poor/Fair |
| **Jeppeson et al, 2023** | Yes | Yes | Not reported | Yes | No | No | Yes | N/A | Yes | N/A | Yes | Yes | Yes | No | Good |
